# Adjunctive ultrasound-guided vagus nerve-targeted pharmacopuncture for refractory fibromyalgia with autonomic symptoms: a case report

**DOI:** 10.3389/fmed.2026.1870404

**Published:** 2026-08-25

**Authors:** Ju-Hyun Lee, Young-Ung Lee, Tan-Hyuk Choi, Suji Lee, Seunghoon Lee

**Affiliations:** 1 Department of Medical Support, Imsil-gun Medical Center, Imsil, Republic of Korea; 2 The Academy of Korean Medicine Clinical Anatomy, Seoul, Republic of Korea; 3 Department of Acupuncture and Moxibustion, Kyung Hee University College of Korean Medicine, Kyung Hee University Medical Center, Seoul, Republic of Korea

**Keywords:** autonomic nervous system, case report, fibromyalgia, heart rate variability, pharmacopuncture, vagus nerve

## Abstract

Fibromyalgia (FM) is a clinical syndrome characterized by chronic widespread pain and various functional symptoms. Although multiple treatments have been proposed for FM, therapeutic options remain limited for patients who do not respond adequately to conventional treatments. This case report describes a woman in her late 50s with refractory FM who received ultrasound-guided vagus nerve-targeted pharmacopuncture (UGP) as an adjunct to multimodal Korean medicine treatment. The patient presented with widespread pain, xerostomia, dyspepsia, headache, palpitations, and insomnia. Multimodal treatment included manual acupuncture, electroacupuncture, routine pharmacopuncture, wet cupping, and herbal medicine, with two additional UGP sessions administered during the treatment period. During the treatment course, the patient’s pain intensity decreased from 7 to 2 on the numeric rating scale, accompanied by improvements in sleep, fatigue, anxiety, depression, and patient-reported autonomic symptoms. Heart rate variability parameters also changed between baseline and the 3-month follow-up. No serious or persistent procedure-related adverse events were observed. However, because UGP was administered as part of a multimodal treatment program and HRV was assessed at only two time points, the specific contribution of UGP to the clinical and physiological changes could not be determined. This case provides a hypothesis-generating observation that warrants further investigation of vagus nerve-targeted interventions in FM.

## 1 Introduction

Fibromyalgia (FM) is a complex clinical syndrome characterized by chronic widespread pain, typically associated with fatigue, non-restorative sleep, cognitive disturbances (often termed ‘fibro-fog’), and impaired concentration. Although the precise etiology of FM remains unclear, a wide range of precipitating factors, including chronic stress, emotional trauma, acute illness, physical injury, and surgery, have been implicated in its onset (1–3). Despite the increasing prevalence of FM, estimated at 2–4% of the global population, managing refractory cases remains a significant clinical challenge owing to the limited efficacy and side effects of conventional pharmacological treatments (4–6).

Dysregulation of the autonomic nervous system, particularly sympathetic nervous system dysfunction, and alterations in the hypothalamic–pituitary–adrenal (HPA) axis have been implicated in the pathophysiology of FM (7, 8). Persistent stressors can trigger chronic sympathetic overactivation and a concomitant reduction in vagal tone, further exacerbating neuroinflammation and central sensitization (9). The vagus nerve participates in autonomic regulation and cholinergic anti-inflammatory signaling, which regulates systemic inflammation and pain processing. Consequently, vagal dysfunction can precipitate pain-related autonomic symptoms and contribute to the complex clinical presentation of FM (10, 11).

Reports indicate that over 50% of patients with FM present clinically significant autonomic symptoms (1, 12). Puri and Lee (13) evaluated the extent of autonomic dysfunction in FM using the Composite Autonomic Symptom Score (COMPASS-31) questionnaire and confirmed a positive correlation between the degree of autonomic impairment and disease severity, as quantified by the Fibromyalgia Impact Questionnaire-Revised (FIQR). These findings suggest that autonomic dysfunction may be a key factor contributing to the functional decline and diminished quality of life in patients with FM.

Various vagus nerve stimulation (VNS) approaches have been implemented to manage chronic pain and autonomic dysfunction. In this context, there has been a growing interest in techniques that allow precise and direct modulation of the vagus nerve.

Ultrasound-guided pharmacopuncture is an injection-based Korean medicine intervention in which a pharmacopuncture solution is administered under real-time ultrasound guidance. In the present report, ultrasound-guided vagus nerve-targeted pharmacopuncture (UGP) refers specifically to the perivagal administration of diluted Hominis placenta extract under ultrasound guidance. The procedure enables direct visualization of the cervical vagus nerve, adjacent vascular structures, needle trajectory, and injectate distribution. During injection, fluid spread around the vagus nerve may separate adjacent tissue planes. Lam et al. (14) reported ultrasound-guided vagus nerve hydrodissection with 5% dextrose in water (D5W) and proposed that its effects might involve mechanical separation of the nerve from surrounding tissues as well as the pharmacological effects of D5W. However, neither pre-existing vagus nerve entrapment nor adhesion was objectively demonstrated in that report.

Evidence regarding ultrasound-guided perivagal injection procedures for FM remains limited, with only a previous case report describing the use of D5W (14). To our knowledge, no previous case report has described the use of Hominis placenta-based UGP as an adjunctive intervention in refractory FM with concurrent assessment of multidimensional clinical outcomes and HRV parameters. Therefore, we report the clinical course of a patient with refractory FM who received two adjunctive UGP sessions during ongoing multimodal Korean medicine treatment. Changes in pain, multidimensional patient-reported outcomes, and HRV parameters were descriptively evaluated. This case report was prepared according to the CARE reporting guidelines.

## 2 Case

### 2.1 Case presentation

A woman in her late 50s presented to the Department of Korean Medicine at Kyung Hee University Korean Medicine Hospital with chronic widespread pain and associated autonomic symptoms. Her symptoms had first developed approximately 3 years earlier following an excessive occupational workload and psychological stress.

She reported severe widespread myalgia involving the neck, shoulders, gluteal region, pelvic region, and bilateral thighs. Associated symptoms included xerostomia, dyspepsia, cephalic pressure accompanied by cognitive clouding (often termed “fibro-fog”), palpitations occurring three to four times daily, insomnia, cold sensations in the hands and feet, paresthesia of the extremities, fatigue, lethargy, and depressive symptoms. She also reported oral and labial pain, a burning sensation at the tip of the tongue, early satiety, nausea, and acid reflux.

Approximately 2 years before presentation, she visited a local hospital and was diagnosed with FM based on clinical findings. Sjögren syndrome was also evaluated using salivary gland assessment and comprehensive serological testing, which reportedly revealed no findings suggestive of the disease.

Over the following 2 years, she received multiple pharmacological treatments, including nonsteroidal anti-inflammatory drugs, pregabalin, nortriptyline, a standardized multi-herbal extract containing *Clematidis Radix*, *Trichosanthis Radix*, and *Prunellae Spica*, and *Vitis vinifera* seed extract. She also underwent extracorporeal shock wave therapy, functional intramuscular stimulation, and physical therapy, without meaningful improvement. Duloxetine and milnacipran were subsequently prescribed but discontinued because of tachycardia.

Approximately 6 months before presentation, her symptoms worsened following renewed psychological stress, particularly the palpitations, cephalic pressure, and cognitive clouding. Laboratory testing, echocardiography, and 24-h Holter monitoring performed at the cardiology department of a local hospital revealed no clinically significant abnormalities. At presentation, she was taking tramadol/acetaminophen for pain and zolpidem for insomnia.

### 2.2 Patient diagnosis

The patient was diagnosed with FM according to the 2016 revised diagnostic criteria of the American College of Rheumatology (15). At the initial visit, the Widespread Pain Index was 10, and the Symptom Severity Scale score was 10. Generalized pain was present in at least four of the five body regions, and the symptoms had persisted for more than 3 years.

At baseline, the patient’s blood pressure was 115/75 mmHg, heart rate was 95 beats/min, and respiratory rate was 26 breaths/min. Musculoskeletal examination revealed tenderness in various areas, including posterior neck, bilateral shoulders, back, lower back, right arm, gluteal muscles, and calves. While movement of the cervical spine and shoulders exacerbated the pain, the symptoms did not follow a focal neuromuscular pattern. Neurological examination revealed no focal neurological deficits; motor strength in the lower extremities was within the normal range, and no pathological findings were detected during the light touch sensation and deep tendon reflex tests. These findings suggested widespread chronic pain in the absence of neurological compromise.

### 2.3 Patient assessment

**Figure 1:**
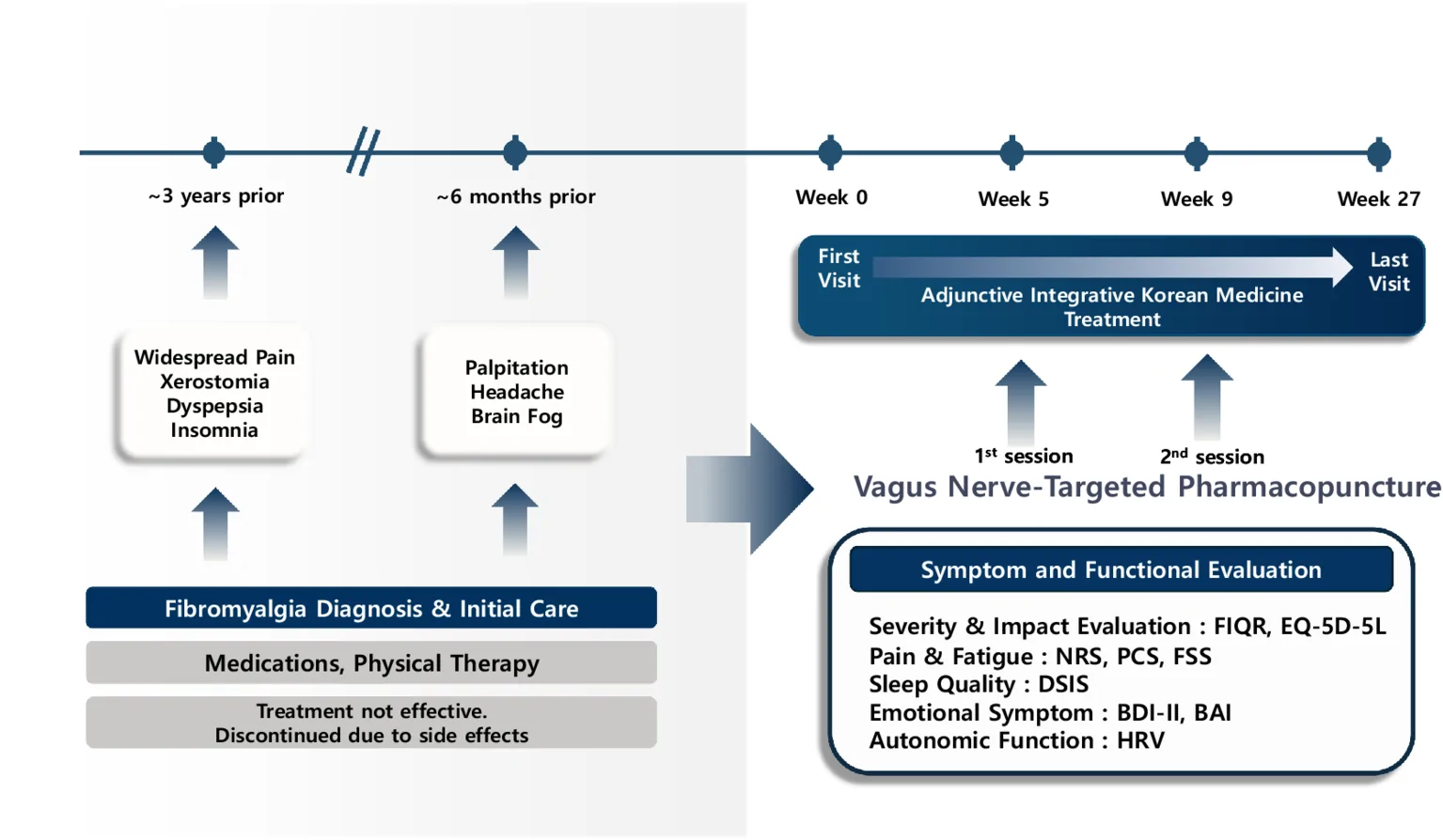
Clinical timeline of symptom onset, prior treatments, and the treatment course. The left panel illustrates the onset of fibromyalgia symptoms and prior treatment history, including medications and physical therapy. The right panel shows the treatment course, including the timing of ultrasound-guided vagus nerve-targeted pharmacopuncture (UGP) sessions (arrows) and assessment time points. BAI, Beck Anxiety Inventory; BDI-II, Beck Depression Inventory-II; DSIS, Daily Sleep Interference Scale; EQ-5D-5L, EuroQol-5 Dimension 5-Level Questionnaire; FIQR, Fibromyalgia Impact Questionnaire-Revised; FSS, Fatigue Severity Scale; HRV, heart rate variability; NRS, Numeric Rating Scale; PCS, Pain Catastrophizing Scale.

Pain intensity was assessed at each visit using an 11-point Numeric Rating Scale (NRS) (16). To evaluate the multidimensional impact of FM and related symptoms, the following patient-reported outcome measures were administered: the validated Korean version of the FIQR (17) to evaluate the impact of FM, the validated Korean version of the Pain Catastrophizing Scale (PCS) (18) to assess pain-related catastrophizing, Daily Sleep Interference Scale (DSIS) (19) to evaluate sleep disturbance, Fatigue Severity Scale (FSS) (20) to assess fatigue severity, the validated Korean version of the Beck Depression Inventory-II (BDI-II) (21) to assess depressive symptoms, the validated Korean version of the Beck Anxiety Inventory (BAI) (22) to assess anxiety level and the Korean version of the EuroQol-5 Dimension 5-Level questionnaire (EQ-5D-5L) (23) to assess quality of life. Autonomic nervous system function was evaluated using HRV analysis (SA-3000; Medicore Co., Ltd., Republic of Korea). HRV measurements were performed after the patient rested quietly for approximately 5 min in a supine position. All measurements were conducted during the morning session before treatment. On the day of assessment, the patient was instructed to refrain from caffeine intake and vigorous physical activity, which could acutely elevate the heart rate. Assessments were performed at two time points: baseline (week 0) and follow-up (approximately 3 months later). The patient’s major symptom course, previous treatment, and treatment timeline at our hospital are summarized in Figure 1.

### 2.4 Therapeutic interventions

A total of 21 treatment sessions were conducted over approximately 6 months. At each visit, the patient received an individualized multimodal Korean medicine treatment program consisting of manual acupuncture, 2-Hz electroacupuncture, routine Hominis placenta pharmacopuncture at symptomatic musculoskeletal sites, and wet cupping therapy. Herbal medicine was administered during the initial 34 days of treatment. The patient received Kracie Kamikihito Extract Fine Granules (Kracie Co., Ltd., Tokyo, Japan), corresponding to Gami-guibi-tang in Korean medicine, at a dose of one packet three times daily. The detailed composition and administration schedule are provided in Supplementary Table 1.

UGP was introduced as an exploratory adjunctive intervention with the aim of modulating vagal or autonomic function and addressing persistent pain and autonomic symptoms. In this report, UGP refers to the ultrasound-guided administration of a diluted Hominis placenta preparation around the cervical vagus nerve using a perineural fluid-distribution technique. The procedure was performed by a board-certified Korean medicine specialist in acupuncture and moxibustion, who had more than 15 years of clinical experience and more than 7 years of experience performing ultrasound-guided procedures.

UGP was added at week 5 because the initial response to the ongoing multimodal treatment program was considered insufficient. A second session was performed at week 9 because residual symptoms persisted and the clinical improvement had plateaued. The timing of the two sessions was based on the patient’s clinical course rather than a predetermined treatment schedule.

#### 2.4.1 Ultrasound-guided vagus nerve-targeted pharmacopuncture

(1) Patient preparation and positioning

The patient was placed in a semi-supine position with the head supported by a low pillow and rotated away from the target side to expose the anterolateral neck. For the first, left-sided procedure, the head was rotated to the right; for the second, right-sided procedure, the head was rotated to the left. A representative external procedural setup using a consenting volunteer is shown in Supplementary Figure 1.

Strict aseptic technique was maintained throughout the procedure. The skin at the injection site was disinfected with a 10% povidone-iodine solution. A small amount of sterile ultrasound gel was applied to the transducer, which was then covered with a sterile sheath while minimizing air entrapment between the transducer and the sheath. A small amount of 10% povidone-iodine solution was also applied to the external surface of the sterile sheath during scanning.

(2) Ultrasound scanning and target localization

A high-frequency linear transducer was placed transversely over the target side of the neck at the level of the C6 transverse process. The carotid artery (CA), internal jugular vein (IJV), and vagus nerve (VN) were identified using cranial and caudal scanning until optimal visualization was achieved. The transducer was adjusted to obtain a clear posterior-to-anterior in-plane needle path toward the perivagal space while avoiding the adjacent vascular structures. The needle was introduced from a posterior skin entry site and advanced under continuous real-time ultrasound visualization.

A dual-mode display was used, with B-mode imaging on one side and color Doppler imaging on the other, to identify the vascular structures along the planned needle trajectory. Before needle insertion, gentle transducer pressure was applied to partially compress the IJV and improve visualization of the procedural field. The needle was advanced under continuous in-plane visualization, with the trajectory adjusted as necessary to avoid the CA and IJV. These measures were used to reduce the risk of inadvertent vascular puncture.

**Figure 2:**
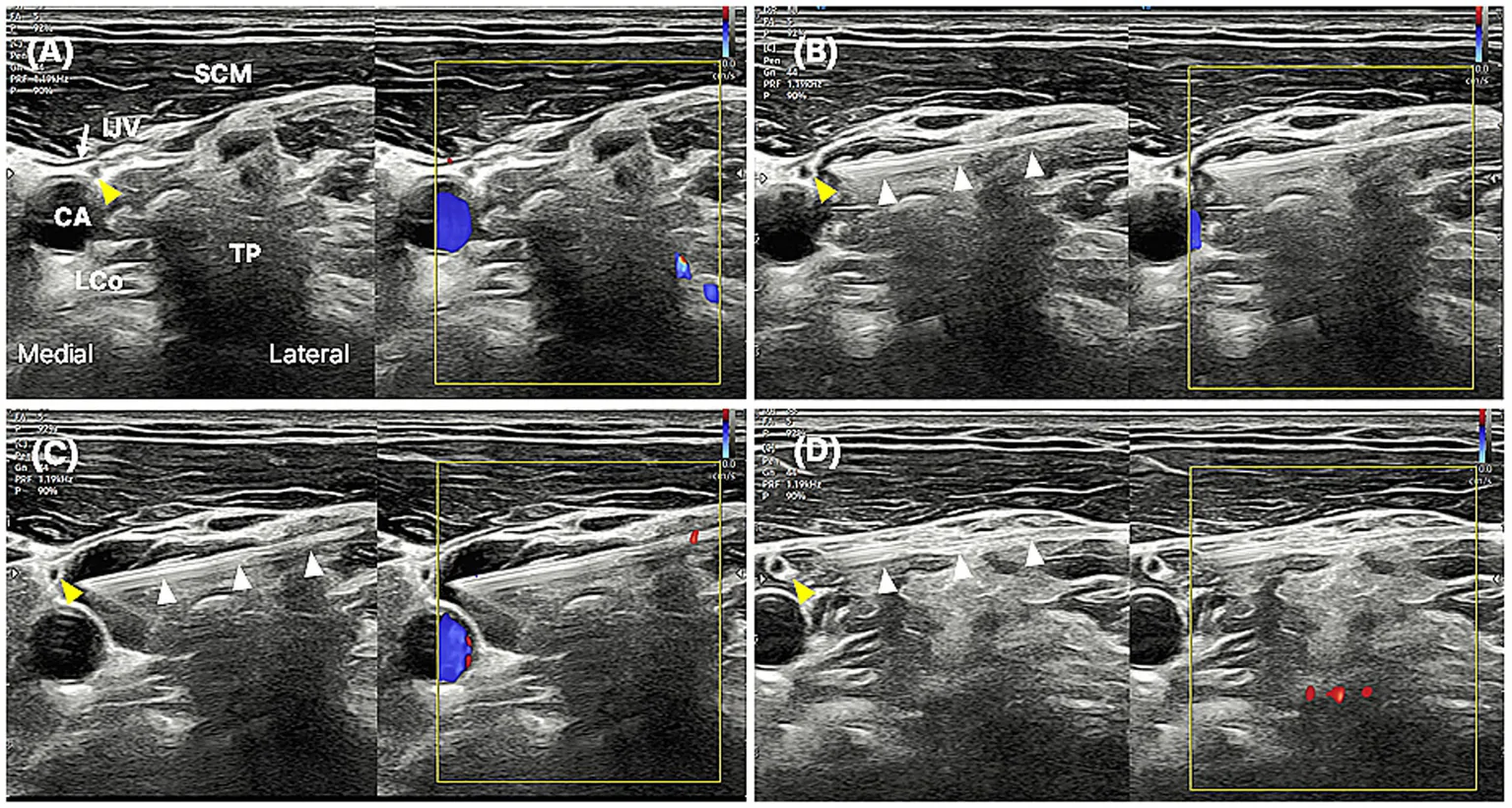
Stepwise procedure of ultrasound-guided vagus nerve-targeted pharmacopuncture. **(A)** Baseline anatomical identification: Transverse ultrasound view at the C6 level showing the vagus nerve (yellow arrowhead) within the carotid sheath. The Carotid Artery (CA), Internal Jugular Vein (IJV, white arrow), Sternocleidomastoid Muscle (SCM), Longus Colli Muscle (LCo), and Transverse Process (TP) are labeled. The IJV appears partially compressed by the transducer. **(B)** Needle approach: The needle (white arrowheads) is advanced using an in-plane technique toward the interface between the vagus nerve (yellow arrowhead) and the carotid artery under continuous ultrasound guidance. **(C)** Initial perivagal fluid spread: Administration of a small volume of injectate produces an anechoic fluid layer adjacent to the vagus nerve (yellow arrowhead), with visible separation of the adjacent tissue planes within the carotid sheath. **(D)** Circumferential perivagal fluid spread: Further administration of the injectate results in fluid distribution around the vagus nerve (yellow arrowhead), producing a circumferential anechoic fluid layer within the carotid sheath. Each panel displays dual-mode imaging, with B-mode imaging on the left and color Doppler imaging on the right, to delineate the adjacent anatomical and vascular structures.

(3) Injectate and injection technique (Figure 2)

For each unilateral procedure, two 2-mL vials of Hominis placenta extract (5 mg dried extract per vial, Girin External Herbal Dispensary, Wonju, Republic of Korea) were diluted with 6 mL of sterile physiological saline to a total volume of 10 mL, corresponding to a final dried-extract concentration of 1 mg/mL. The entire 10-mL volume was administered during each unilateral session. Across the two sessions, four vials were used, corresponding to a total of 20 mg of dried extract.

Each session was performed unilaterally. Because the patient’s symptoms were systemic and no side-specific clinical findings favored either side, the left side was selected first according to the operator’s usual procedural sequence. The right side was treated approximately 4 weeks later to avoid repeating the procedure on the same side. Side selection was not based on a predefined protocol or an anticipated side-specific difference in efficacy. No local anesthetic was administered to avoid introducing an additional pharmacologically active injectate and to preserve immediate patient feedback during needle advancement and fluid administration. Continuous verbal communication was maintained to monitor for unexpected pain, paresthesia, coughing, voice changes, dizziness, or other symptoms.

A 26-gauge, 6-cm needle was advanced using an in-plane approach at an approximate angle of 15° relative to the skin surface. This maneuver facilitated a clearer needle path and reduced the risk of venipuncture. The injectate was administered using a two-stage perivagal injection technique.

First, the needle was introduced approximately 1 cm from the posterior edge of the transducer, with the bevel facing upward, and advanced using a posterior-to-anterior in-plane approach under continuous real-time ultrasound visualization. The needle tip was directed toward the perivagal space within the carotid sheath. As the needle tip approached the intended target, gentle aspiration was performed as an additional precaution, followed by administration of a small test aliquot. The final needle-tip position was confirmed by direct ultrasound visualization adjacent to the vagus nerve and by the appearance of anechoic fluid spread within the perivagal space. A portion of the injectate was then administered incrementally, producing initial perivagal fluid spread and separation of the adjacent tissue planes. Color Doppler imaging was intermittently used to reassess the relationship between the needle trajectory and the adjacent vascular structures.

Second, after confirming initial perivagal fluid spread, the needle was withdrawn slightly along its original path to a position just superficial to the carotid sheath near the medial border of the sternocleidomastoid muscle. The needle was then rotated 180° with the bevel facing downward, and re-advanced toward the anterolateral aspect of the nerve under continuous in-plane visualization. The remaining injectate was administered incrementally to produce further fluid distribution around the vagus nerve and a circumferential anechoic fluid layer within the carotid sheath.

The intended target was the perivagal space within the carotid sheath, and the needle was not intentionally advanced into the prevertebral or interscalene compartments. The planned trajectory was selected to avoid the prevertebral and interscalene regions and the expected locations of the cervical sympathetic chain, phrenic nerve, and brachial plexus. The CA and IJV were mapped using B-mode and color Doppler imaging, and the needle shaft and tip were maintained under continuous in-plane visualization during advancement. Gentle transducer pressure was applied when necessary to partially compress the IJV and improve visualization of the procedural field. The total procedural time was approximately 5–7 min.

(4) Patient communication and post-procedure care

Throughout the procedure, continuous verbal communication was maintained with the patient to reduce anxiety and to monitor discomfort. The patient was informed of each step, including needle advancement and fluid injection.

Following the injection, the needle was withdrawn, and the site was disinfected. Sterile adhesive bandages were also applied. The patient was instructed to rest in the supine position for approximately 20 min for observation. During this period, the patient was monitored for immediate adverse reactions, including bleeding, localized pain, dizziness, and nausea. Vital signs, including heart rate and blood pressure, were measured immediately after the procedure, and no clinically significant abnormalities were observed.

### 2.5 Treatment outcomes

(1) Longitudinal change in pain intensity

**Figure 3:**
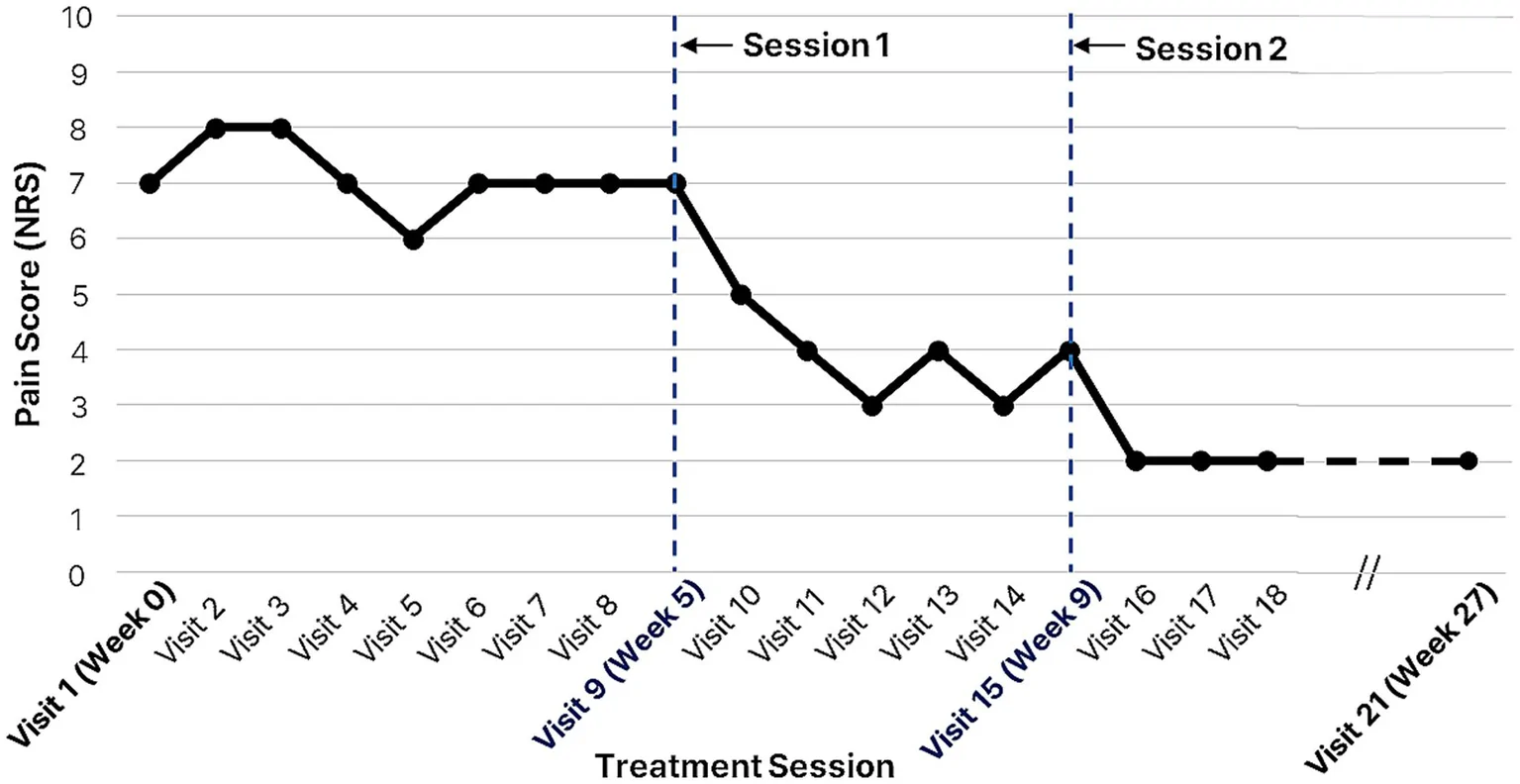
Longitudinal changes in pain intensity measured using the Numeric Rating Scale. Each data point represents the pain score recorded at each treatment visit. Vertical dashed lines indicate the timing of ultrasound-guided vagus nerve-targeted pharmacopuncture sessions (Session 1 and Session 2). The break in the *x*-axis (//) indicates a gap between Visit 18 and Visit 21. NRS, Numeric Rating Scale; UGP, ultrasound-guided vagus nerve-targeted pharmacopuncture.

The patient’s pain intensity, assessed using the NRS, decreased from 7 to 2 during the treatment period. During the initial phase of the multimodal treatment program, the NRS score remained at 7, indicating a limited early response. UGP was introduced at week 5, after which the NRS score gradually decreased during the ongoing multimodal treatment course. A second UGP session was performed at week 9, when residual symptoms persisted and improvement had plateaued; the NRS score subsequently decreased from 4 to 2. A more pronounced decline in NRS scores was observed temporally after the introduction of UGP and was subsequently maintained at a stable, low level throughout the remaining treatment course. However, because all other treatment components were continued concurrently, this temporal association does not establish a specific treatment effect of UGP (Figures 1, 3).

(2) Multidimensional clinical improvement

The clinical effects of treatment are reflected in a broad spectrum of FM-related symptoms. Standardized assessments conducted 3 months post-baseline showed consistent and meaningful improvements (Table 1). The FIQR score decreased from 78 at baseline to 34 at the 3-month follow-up. Anxiety (BAI: 32–19) and depression (BDI-II: 26–19) scores improved markedly, along with a substantial reduction in pain catastrophizing (PCS: 47–25). The EQ-5D-5L index increased from 0.275 to 0.706, indicating a significant improvement in health-related quality of life.

(3) Patient-reported changes in autonomic symptoms

**Table 1:** Clinical outcome measures at baseline and the 3-month follow-up.

Domain	Measure	Baseline	3 months
Disease Severity	FIQR	78	34
Pain Perception	PCS	47	25
Emotional Symptoms	BDI-II	26	19
	BAI	32	19
Sleep Quality	DSIS	8	4
Fatigue	FSS	5.8	3.6
Quality of Life	EQ-5D-5L	0.275	0.706

BAI, Beck Anxiety Inventory; BDI-II, Beck Depression Inventory-II; DSIS, Daily Sleep Interference Scale; EQ-5D-5L, EuroQol-5 Dimension 5-Level questionnaire; FIQR, Fibromyalgia Impact Questionnaire-Revised; FSS, Fatigue Severity Scale; PCS, Pain Catastrophizing Scale.

During the treatment period, the patient reported improvements in xerostomia, palpitations, and dyspepsia. In particular, she reported that the frequency and intensity of palpitations decreased beginning the day after the first UGP session. These symptom changes were assessed through clinical interviews and were not quantified using a standardized autonomic symptom scale.

(4) Changes in HRV parameters

Changes in HRV parameters between baseline and the 3-month follow-up are presented in Table 2. The low-frequency-to-high-frequency (LF/HF) ratio decreased from 5.20 to 1.26. The standard deviation of normal-to-normal intervals (SDNN) increased from 11.08 to 20.32 ms, and the root mean square of successive differences (RMSSD) increased from 4.27 to 12.93 ms. The mean heart rate decreased from 103 to 73 bpm. Because HRV was assessed at only two time points, these findings are descriptive and cannot establish autonomic recovery or vagal modulation attributable to UGP.

(5) Patient perspective

**Table 2:** Heart rate variability parameters at baseline and the 3-month follow-up.

Domain	Measure	Baseline	3 months
Global Autonomic Variability	Mean heart rate (bpm)	103	73
	SDNN (ms)	11.08	20.32
	TP (ms^2^)	131.57 (4.88)	437.46 (6.08)
Parasympathetic Activity	RMSSD (ms)	4.27	12.93
	HF (ms^2^)	11.21 (2.42)	58.44 (4.07)
Sympathovagal Balance	LF (ms^2^)	58.28 (4.07)	73.77 (4.30)
	LF/HF Ratio	5.20	1.26

HF, High-Frequency Power; LF, Low-Frequency Power; LF/HF, Low-Frequency-to-High-Frequency Ratio; RMSSD, Root Mean Square of Successive Differences; SDNN, Standard Deviation of Normal-to-Normal Intervals; TP, Total Power. For frequency domain metrics (TP, HF, LF), absolute power values are expressed in ms^2^, with corresponding natural logarithm-transformed values shown in parentheses.

The patient described seeking treatment at our hospital as a last resort after experiencing adverse effects from conventional medications. She reported that following the first session of UGP, palpitations decreased and bodily tension was noticeably reduced from the next day. She subsequently noted a progressive improvement in widespread pain and overall symptoms throughout the remaining treatment course.

### 2.6 Adverse events

Overall, UGP was well tolerated by the patient. The adverse events observed during the treatment were limited and mild in severity. Specifically, there was a single transient episode of procedural anxiety and an instance of localized pain at the injection site, both of which were self-limiting. No serious adverse events typically associated with cervical VNS, including bradycardia, syncope, hoarseness, or persistent coughing, were observed during the study period. All recorded clinical events resolved within minutes, leaving no lasting sequelae and requiring no additional medical intervention.

## 3 Discussion

This case report describes a patient with treatment-refractory FM who showed improvements in pain and multiple associated symptoms during a six-month multimodal Korean medicine treatment course, in which two sessions of UGP were administered as adjunctive interventions. The patient showed a limited response to multiple pharmacological and non-pharmacological treatments. During the present treatment course, pain intensity, sleep disturbance, fatigue, psychological symptoms, and patient-reported autonomic symptoms improved. HRV parameters also changed between baseline and the 3-month follow-up. However, because UGP was administered concurrently with multiple other interventions and HRV was assessed at only two time points, these clinical and physiological changes cannot be attributed specifically to UGP or interpreted as evidence of autonomic recovery. No serious or persistent procedure-related adverse events were observed.

Autonomic dysregulation is highly prevalent in patients with FM and is recognized as a major contributing factor to the exacerbation of clinical symptoms. The vagus nerve plays a pivotal role in modulating systemic inflammatory and stress responses primarily through the regulation of cortisol secretion and activation of the cholinergic anti-inflammatory pathway. Consequently, vagus nerve dysfunction has been postulated to precipitate pain-related autonomic symptoms (9–11, 24). Given that vagal impairment has been proposed as a central mechanistic component of the FM pathophysiology, there is increasing clinical and scientific interest in neuromodulation-based therapies that directly target the vagus nerve.

VNS is an invasive neuromodulatory therapy involving surgical implantation of electrodes or coils along the cervical vagus nerve to control epileptic seizures refractory to pharmacological treatment. With subsequent technological advancements, noninvasive transcutaneous approaches (e.g., auricular and cervical VNS) have become feasible, expanding VNS’s therapeutic application in various fields, including depression, chronic pain, and inflammatory disorders (25).

The previous report using 5% dextrose provides a procedural precedent for ultrasound-guided perivagal injection. However, D5W and Hominis placenta extract are biologically and clinically distinct injectates; therefore, the pharmacological or physiological effects proposed for D5W cannot be extrapolated to Hominis placenta pharmacopuncture. In the present case, fluid administration produced perivagal distribution and separation of adjacent tissue planes, but no objective evidence of pre-existing vagus nerve entrapment or adhesion was identified. Accordingly, the respective contributions of perivagal targeting, injectate volume, fluid distribution, and Hominis placenta extract remain uncertain.

The UGP procedure may be conceptualized as a multimodal intervention involving mechanical, needle-related neuromodulatory, and pharmacological components. First, administration of a relatively large fluid volume around the vagus nerve produced circumferential perineural spread and separation of the adjacent tissue planes. Such fluid distribution may theoretically alter local perineural tissue relationships or reduce mechanical restriction; however, because no objective evidence of vagus nerve entrapment, adhesion, or compression was identified, it should not be interpreted as confirmed decompression of the vagus nerve. Second, ultrasound-guided needle placement and fluid administration in close proximity to the vagus nerve may have provided localized mechanical stimulation and may theoretically influence vagal-related autonomic signaling. The vagus nerve is involved in autonomic regulation and the cholinergic anti-inflammatory pathway (9, 11, 14); however, the present case did not directly assess neural activation or vagal signaling. Accordingly, the observed HRV changes cannot establish that UGP directly modulated or normalized vagal function. Third, Hominis placenta extract contains biologically active constituents that have been proposed to exert anti-inflammatory or tissue-supportive effects (26). These properties may theoretically influence the local perineural environment. Nevertheless, its pharmacological effects in the cervical perivagal space have not been established, and its contribution cannot be separated from those of needle placement, perivagal targeting, and injectate volume. Thus, the proposed mechanisms should be regarded as exploratory and hypothesis-generating.

A recent case report by Lam et al. (14) described clinical improvement following ultrasound-guided vagus nerve hydrodissection with D5W. The present case used a biologically and clinically distinct injectate and provides additional descriptive clinical data; therefore, the mechanisms or effects proposed for D5W cannot be extrapolated to Hominis placenta-based UGP. Furthermore, our study provides additional descriptive physiological data from HRV measurements at baseline and follow-up, including the LF/HF ratio, alongside a broader array of multidimensional assessments, such as the PCS, FSS, and BAI/BDI-II, compared with the resting heart rate and ECG monitoring reported in the previous study. Consequently, this case report provides a hypothesis-generating observation that supports further investigation of ultrasound-guided vagus nerve-targeted interventions for the complex somatic and autonomic manifestations of FM.

The present case report had several important limitations. First, as a single case report, it inherently provides low-level evidence and has limited generalizability. Second, because the injectate consisted of diluted Hominis placenta extract, it was not possible to distinguish the potential pharmacological contribution of the extract from the effects of needle placement, perivagal targeting, injected volume, and fluid-mediated separation of the adjacent tissue planes. Third, the concurrent application of multiple therapeutic modalities, including herbal medicine, manual acupuncture, electroacupuncture, routine Hominis placenta pharmacopuncture, and wet cupping, precluded isolation of the specific contribution of UGP or any other individual intervention to the observed clinical improvement. In addition, the patient had experienced multiple previous treatment failures and regarded treatment at our hospital as a “last resort,” which may have increased treatment expectancy and contextual responses. Treatment was also initiated during a period of marked symptom exacerbation following renewed psychological stress; therefore, spontaneous stabilization, natural symptom fluctuation, regression to the mean, and nonspecific time effects may have contributed to the subsequent improvement. These alternative explanations cannot be distinguished from the combined and cumulative effects of the multimodal treatment program or the adjunctive UGP procedures in this single case. Fourth, the timing and number of UGP sessions were determined according to the patient’s clinical course and the treating clinician’s judgment rather than a standardized protocol. Therefore, the optimal treatment interval and number of sessions remain unknown. Fifth, given the anatomical complexity of the cervical region, the possibility of inadvertent stimulation or injury of adjacent nontarget structures cannot be fully excluded, and the procedure requires a high level of technical expertise. Sixth, HRV measurements were obtained at only two time points and may have been influenced by the psychological state, sleep quality, and other contextual factors, limiting their physiological interpretation. Accordingly, whether UGP influenced autonomic function cannot be determined from the present HRV findings.

Collectively, these limitations restrict causal inferences and underscore the need for controlled studies to better delineate the therapeutic contributions and mechanisms of UGP. Future research should incorporate controlled designs comparing UGP with Hominis placenta extract versus an equivalent-volume saline injection using the same ultrasound-guided perivagal technique. Such studies may help distinguish the potential pharmacological contribution of the extract from the effects of needle placement, perivagal targeting, injectate volume, and fluid-mediated tissue-plane separation. Large-scale longitudinal studies are needed to clarify the potential mechanisms and assess the generalizability of the observed therapeutic responses.

From a procedural perspective, UGP is performed in an anatomically confined region adjacent to the carotid artery, the internal jugular vein, and other critical neurovascular structures. This proximity entails the theoretical risk of unintended vascular or neural stimulation, underscoring the importance of substantial operator expertise and meticulous real-time ultrasound guidance. Accordingly, the development of standardized procedural protocols is important to ensure safety and facilitate the broader clinical application of this technique.

Despite these limitations, this case has several clinically significant implications. First, the patient who was refractory to multiple conventional therapies showed subsequent improvements in pain and autonomic symptoms after UGP was introduced. Although causal inference remains limited owing to the multimodal treatment context, the temporal association between UGP introduction and symptom improvement is noteworthy. Second, improvements were observed not only in subjective pain and impact measures but also in HRV indices, suggesting a possible modulation of autonomic imbalance, a hypothesized core pathology, rather than merely providing transient symptomatic relief. This interpretation should be considered exploratory. Third, this case suggests that vagus nerve-targeted neuromodulatory or perineural injection approaches may represent potential therapeutic options for highly refractory conditions such as FM. Collectively, these observations provide hypothesis-generating support for further evaluation of vagus nerve-targeted interventions in treatment-resistant FM.

## Data Availability

The datasets presented in this article are not readily available because this is a case report. Requests to access the datasets should be directed to Seunghoon Lee, kmdoctorlee@khu.ac.kr.
