# Supplementary material for: Adjunctive ultrasound-guided vagus nerve-targeted pharmacopuncture for refractory fibromyalgia with autonomic symptoms: a case report
Source: Front Med (Lausanne). 2026 Aug 25;13:1870404. doi: 10.3389/fmed.2026.1870404 (PMC13550960; doi:10.3389/fmed.2026.1870404)
Supplement: Supplementary file 1 [file Table_1.DOCX]

Supplementary Table 1. Summary of Concomitant Korean Medicine Treatments

| Intervention | Treatment details | Dose and parameters | Frequency and duration |
| --- | --- | --- | --- |
| Manual acupuncture | Performed in the prone position. Local symptomatic regions included the neck, shoulders, lower back, and gluteal region. Distal points included unilateral PC6, HT7, and SP6. | Sterile disposable needles, 0.25 × 40 mm; insertion depth, 2–10 mm; manual stimulation until deqi; retention time, 15 min. | At each treatment visit; 21 sessions |
| Electroacupuncture | Applied between PC6 and HT7. | 2 Hz for 15 min; intensity adjusted until visible muscle twitching was observed. | At each treatment visit; 21 sessions |
| Wet cupping therapy | Applied to symptomatic regions, including the neck, shoulders, and lower back. | Sterile lancet and disposable cups; application time, approximately 5 min. | At each treatment visit; 21 sessions |
| Routine pharmacopuncture | Hominis placenta extract was administered to 6–8 symptomatic sites involving the neck, shoulders, and gluteal region. | 30-gauge needle; total volume, 2 mL; approximately 0.1–0.2 mL per site. | At each treatment visit; 21 sessions |
| Herbal Medicine | Kracie Kamikihito Extract Fine Granules (K-19), corresponding to Gami-guibi-tang. | One 3.75-g packet^*^ orally three times daily, 30 min after meals. | Days 1–34; total duration, 34 days |

^*^ Each 3.75-g packet contained dried extract prepared from the following crude herbal materials: Ginseng Radix, Atractylodis Rhizoma Alba, Poria Sclerotium, Zizyphi Semen, Longan Arillus, and Bupleuri Radix, 1.5 g each; Astragali Radix, Angelicae Gigantis Radix, and Gardeniae Fructus, 1.0 g each; Polygalae Radix and Zizyphi Fructus, 0.75 g each; Glycyrrhizae Radix and Aucklandiae Radix, 0.5 g each; and Zingiberis Rhizoma, 0.25 g.
