# Supplementary material for: Adjunctive ultrasound-guided vagus nerve-targeted pharmacopuncture for refractory fibromyalgia with autonomic symptoms: a case report
Source: Front Med (Lausanne). 2026 Aug 25;13:1870404. doi: 10.3389/fmed.2026.1870404 (PMC13550960; doi:10.3389/fmed.2026.1870404)
Supplement: Supplementary file 2 [file Table_2.DOCX]

**Supplementary Figure 1. Representative patient positioning and external procedural setup for ultrasound-guided vagus nerve-targeted pharmacopuncture.**
The photograph shows a consenting volunteer, who was not the patient described in this case report, in a semi-supine position with the head rotated to the right to expose the left anterolateral neck. A high-frequency linear ultrasound transducer was positioned transversely over the left side of the neck. The needle was positioned at the posterior skin entry site to demonstrate the intended in-plane posterior-to-anterior approach. Written informed consent for publication of the photograph was obtained from the volunteer.
