# Supplementary material for: Adjunctive ultrasound-guided vagus nerve-targeted pharmacopuncture for refractory fibromyalgia with autonomic symptoms: a case report
Source: Front Med (Lausanne). 2026 Aug 25;13:1870404. doi: 10.3389/fmed.2026.1870404 (PMC13550960; doi:10.3389/fmed.2026.1870404)
Supplement: Supplementary file 3 [file Table_3.DOCX]

**CARE Checklist**

*Adjunctive Ultrasound-Guided Vagus Nerve-Targeted Pharmacopuncture for Refractory Fibromyalgia with Autonomic Symptoms: A Case Report*

| **Topic** | **Item** | **CARE Checklist Item Description** | **Reported on Line / Section** |
| --- | --- | --- | --- |
| **Title** | 1 | The diagnosis or intervention of primary focus followed by the words "case report" | Title (lines 1–3) |
| **Key Words** | 2 | 2 to 5 key words that identify diagnoses or interventions in this case report, including "case report" | Keywords (lines 15–16) |
| **Abstract** | 3a | Introduction: What is unique and why is it important? | Abstract (lines 18–35) |
|  | 3b | The patient’s main concerns and important clinical findings | Abstract (lines 18–35) |
|  | 3c | The main diagnoses, therapeutic interventions, and outcomes | Abstract (lines 18–35) |
|  | 3d | Conclusion: What is the main “take-away” lesson(s) from this case? | Abstract, final sentence (lines 33–35) |
| **Introduction** | 4 | One or two paragraphs summarizing why this case is unique with medical literature references | Introduction (lines 36–82) |
| **Patient Information** | 5a | De-identified demographic and other patient-specific information | Section 2.1, Case Presentation, paragraph 1 (lines 84–87) |
|  | 5b | Main concerns and symptoms of the patient | Section 2.1, Case Presentation, paragraphs 1–2 (lines 84–96) |
|  | 5c | Medical, family, and psychosocial history including diet, lifestyle, and genetic information whenever possible, including relevant comorbidities and past interventions and their outcomes | Section 2.1, Case Presentation, paragraphs 1 and 3–5 (lines 84–105) |
| **Clinical Findings** | 6 | Describe the relevant physical examination (PE) and other significant clinical findings | Section 2.2, Patient Diagnosis (lines 106–121) |
| **Timeline** | 7 | Depict important dates and times in this case (a figure or table is strongly recommended) | Figure 1; Section 2.3, Patient Assessment (lines 122–141) |
| **Diagnostic Assessment** | 8a | Diagnostic methods (such as PE, laboratory testing, imaging, surveys, and questionnaires) | Sections 2.1–2.3 (lines 84–141) |
|  | 8b | Diagnostic challenges (such as access to or financial or cultural) | Not applicable |
|  | 8c | Diagnostic reasoning including other diagnoses considered | Section 2.1, paragraph 3 (Sjögren syndrome evaluated; lines 97–99) |
|  | 8d | Prognostic characteristics (such as staging in oncology) where applicable | Not applicable |
| **Therapeutic Intervention** | 9a | Types of intervention (such as pharmacologic, surgical, preventive, self-care) | Sections 2.4 and 2.4.1, Therapeutic Interventions (lines 142–243) |
|  | 9b | Administration of intervention (such as dosage, strength, duration, and frequency) | Sections 2.4 and 2.4.1 (lines 142–243); Supplementary Table 1 |
|  | 9c | Changes in the intervention (with rationale) | Section 2.4 (UGP added at weeks 5 and 9; lines 157–162); Section 2.4.1 (side selection; lines 188–197) |
| **Follow-up and Outcomes** | 10a | Clinician-assessed and patient-assessed outcomes (when appropriate) | Section 2.5, Treatment Outcomes, subsections 1–4 (lines 244–280); Tables 1–2; Figure 3 |
|  | 10b | Important follow-up diagnostic and other test results | Section 2.5(4), Changes in HRV Parameters (lines 269–280); Table 2 |
|  | 10c | Intervention adherence and tolerability (how was this assessed?) | Section 2.4 (21 completed treatment sessions; lines 142–156); Section 2.6, Adverse Events (lines 286–293) |
|  | 10d | Adverse and unanticipated events | Section 2.6, Adverse Events (lines 286–293) |
| **Discussion** | 11a | Strengths and limitations in your approach to this case | Discussion, limitations and clinical implications (lines 350–408) |
|  | 11b | Discussion of the relevant medical literature | Discussion (lines 294–349) |
|  | 11c | The rationale for conclusions (including assessment of possible causes) | Discussion, mechanistic interpretation and alternative explanations (lines 321–385) |
|  | 11d | The primary “take-away” lessons from this case report | Discussion, final paragraph (lines 394–408) |
| **Patient Perspective** | 12 | The patient should share their perspective on the treatments they received | Section 2.5(5), Patient Perspective (lines 281–285) |
| **Informed Consent** | 13 | Did the patient provide informed consent? Please provide if requested | Ethics Statement (lines 411–416) |

*This checklist was updated in accordance with the CARE guidelines (https://www.care-statement.org/checklist).*
